# Primary extraskeletal intradural Ewing sarcoma with acute hemorrhage: a case report and review of the literature

**DOI:** 10.1186/s13256-024-04384-8

**Published:** 2024-03-09

**Authors:** HusamEddin Salama, Lila H. Abu-Hilal, Mayar Idkedek, Abdalwahab Kharousha, Mohand Abulihya, Hafez Nimer

**Affiliations:** 1 Medical Research Club, Faculty of Medicine, Al-Quds University, Jerusalem, Palestine; 2 Department of Neurosurgery, Al-Istishari Arab Hospital, Ramallah, West Bank Palestine; 3 Department of Pathology, Al-Istishari Arab Hospital, Ramallah, West Bank Palestine

**Keywords:** Ewing sarcoma, Extra-skeletal, Intradural, Intratumoral hemorrhage, Tumor, Chemotherapy, Radiotherapy

## Abstract

**Background:**

Spinal cord tumors present a challenge in diagnosis and treatment due to their varied histopathological characteristics. While Ewing sarcoma is a rare malignant tumor typically originating from skeletal bone, cases of primary intradural extraskeletal Ewing sarcoma are exceptionally rare. The similarity of its presentation to other spinal tumors further complicates its identification and management.

**Case presentation:**

We report a case of a 58-year-old Palestinian male with intradural extraskeletal lumbar Ewing sarcoma. The patient initially presented with lower back pain and bilateral S1 radiculopathy, with more severe symptoms on the left side. Magnetic resonance imaging revealed a 7 cm oval-shaped mass with homogeneous contrast enhancement, obstructing the spinal canal from L3/L4 to L5/S1 levels. Initially, a myxopapillary ependymoma was suspected, but the patient’s sensory and motor functions suddenly deteriorated during hospitalization. Repeat magnetic resonance imaging indicated heterogeneous contrast enhancement, indicating acute intratumoral hemorrhage. Consequently, the patient underwent emergent L3–L5 laminotomy, with successful gross total resection of the tumor. Histopathological and immunohistochemical analyses confirmed the diagnosis of intradural extraskeletal Ewing sarcoma. Adjuvant therapy was administered to minimize the risk of local recurrence or distant metastasis. A systematic review of relevant literature, along with retrospective analysis of medical records, operative reports, radiological studies, and histopathological findings of similar cases, was also conducted.

**Conclusions:**

Intradural extraskeletal Ewing sarcoma is an infrequently encountered condition in adult patients, emphasizing the importance of considering it in the differential diagnosis of spinal tumors. Surgeons must possess a comprehensive understanding of this rare entity to ensure accurate staging and optimal management, particularly in the early stages when prompt intervention may improve prognosis.

## Introduction

Ewing sarcoma, which was first described by James Ewing in 1921 [1], is an extremely aggressive, undifferentiated, primitive, small blue round cell malignant tumor of the bones and other soft tissues [2]. It arises from the neuroectodermal cells. Although rare, Ewing sarcoma is considered the second most common primary bone malignancy affecting children and young adults, with slight male predominance. It accounts for about 2% of all childhood malignancies with an incidence of about 2.8 cases per million for people under the age of 19 years [3]. It classically presents as enlarging, painful, lytic bone lesion in the diaphysis of long bones and pelvic flat bones and it usually metastasizes to the lungs, skeletal system, and bone marrow, which are usually found at the time of the diagnosis. There is a unique pattern of chromosomal translocations in the family of Ewing sarcoma tumors. All of them hold great importance in the process of diagnosis and management.

In 1969, Tefft firstly described a tumor with similar histological features in the paravertebral region [4]. That was the first documentation of an extraosseous form of Ewing sarcoma. The extraskeletal subtype of Ewing sarcoma usually presents in the lower extremities, chest wall, and paravertebral region. Less commonly, it can occur in the retroperitoneal region, pelvis, upper limb, head, and neck [5]. The classic presentation of these patients is mass in deep soft tissue with local pain at the affected site but with no signs of an inflammatory reaction at the surface of the affected region [6]. It has a low incidence rate and accounts for 1.1% of all malignant soft tissue tumors, which is ten times less than that of osseous Ewing sarcoma [7]. Unlike Ewing sarcoma of the bone, extraskeletal Ewing sarcoma has a bimodal distribution peaking in those who are < 5 years and > 35 years old, has no relation to sex, and both classes of Ewing sarcoma are more dominant among the white population [8].

Primary spinal cord tumors are a relatively rare form of cancer, accounting for about 3% of all central nervous system tumors [9]. The incidence rate of these tumors varies depending on the specific type and location of the tumor. They can be classified based on their anatomic location in relation to the dura mater and spinal cord into three categories: epidural tumors, intradural extramedullary tumors, or intradural intramedullary [10]. Intradural extramedullary tumors are located inside the dura but outside the spinal cord. They can arise from the nerve roots, the meninges (the membranes that surround the spinal cord), or other structures within the spinal canal. Intradural extramedullary tumors can also compress the spinal cord or nerve roots, leading to symptoms such as pain, weakness, or numbness. Extraskeletal intradural extramedullary Ewing sarcoma is extremely rare. Herein, we present a case of a 58-year-old male patient with primary extraskeletal intradural extramedullary Ewing sarcoma in the lumbar region with acute intratumoral hemorrhage, which is an uncommon presentation for intradural extraskeletal Ewing sarcoma (IEES), with a review of the pertinent literature.

## Case presentation

A 58-year-old Palestinian male, with free past medical history, presented to our hospital with complaints of mild, tolerable lower back pain of 2 months duration, which was responding well to celecoxib. Later on, the pain was associated with bilateral numbness down the back of his legs, which was more prominent on the left side, consistent with S1 radiculopathy. Thus, the patient sought medical advice and underwent a lumbosacral spine computed tomography (CT) scan, which was unremarkable, so the patient continued on celecoxib with no improvement. Two weeks prior to admission to our hospital, the patient’s pain increased dramatically with increasing radiculopathy and numbness not specific to any dermatome, with distal weakness of the lower limbs more prominent on the left side and affected his daily life activities. In addition he had a history of sphincter dysfunction with a description of constipation and incontinence.

**Fig. 1:**
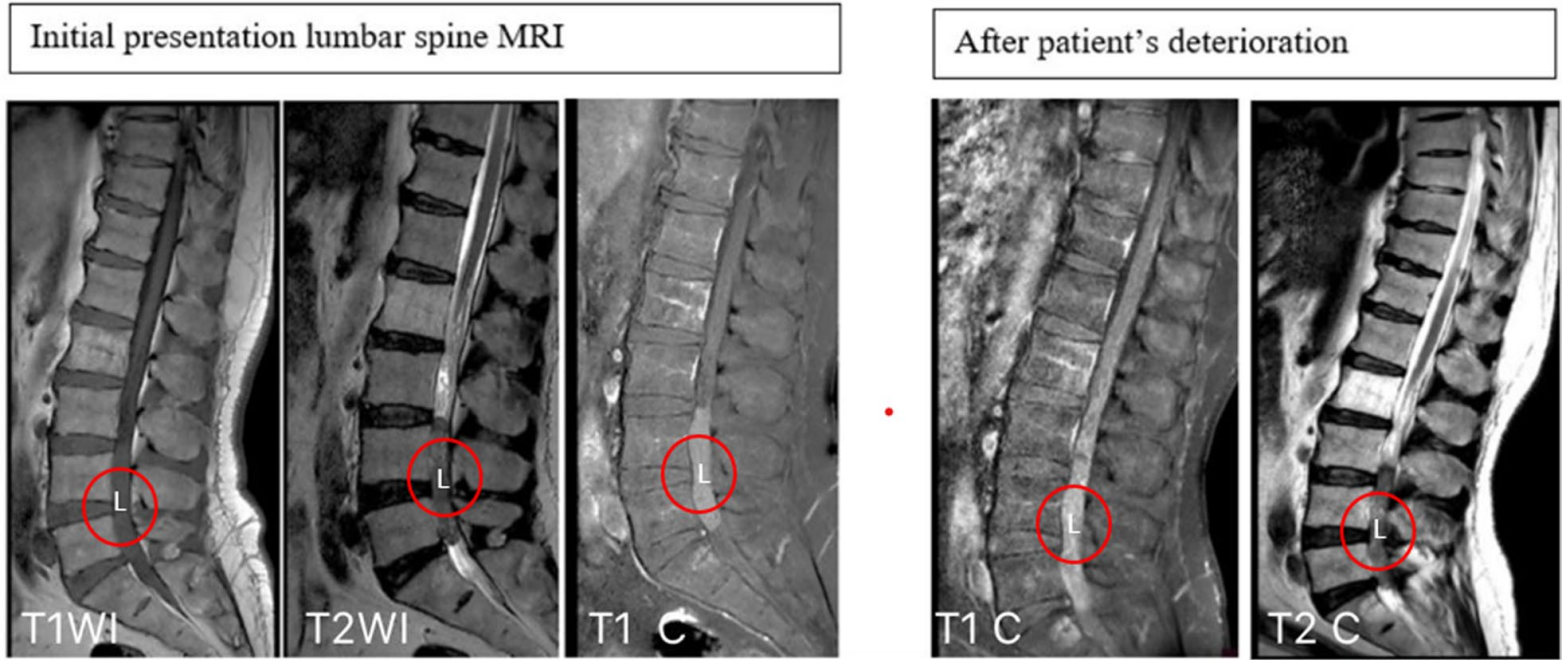
Sagittal lumber MRI images at the initial presentation and after the deterioration of the patient’s status. At the initial presentation, T1 and T2 noncontrasted sequence showing intermediate T1, low T2 signal-intensity intradural/ extramedullary lesion extending from the lower aspect of L3 to L5–S1 disc level, while sagittal T1 contrasted image showing diffuse homogenous enhancement of the lesion. After the deterioration of patient’s symptoms, sagittal T1- and T2-contrasted images showing change in intensity in T2 and change in enhancement with contrast to a heterogenous pattern, suggestive of intratumoral hemorrhage. L: lesion; T1WI: T1 MRI image without contrast; T2WI: T2 MRI image without contrast; T1 C: T1 MRI image with contrast; T2 C: T2 MRI image with contrast (red circle indicates the letter L)

Neurological examination showed positive bilateral straight leg raise test, bilateral hypoesthesia on S1 dermatome, positive patellar tendon reflex, and decreased Achilles tendon reflex. Babinski and Chaddock reflexes were negative on both sides. The manual muscle test score was 3/5 in the left dorsiflexion and plantarflexion of the foot, while it was 4/5 on the right foot. All proximal muscles were intact. Pre- and post-voiding ultrasounds were done and suggested neurogenic bladder. Accordingly, lumbar spine magnetic resonance imaging (MRI) was done and showed a 7 cm oval-shaped mass obliterating the spinal canal from L3/L4 to L5/S1 levels with homogeneous enhancement on contrast administration as shown in Fig. 1.

**Fig. 2:**
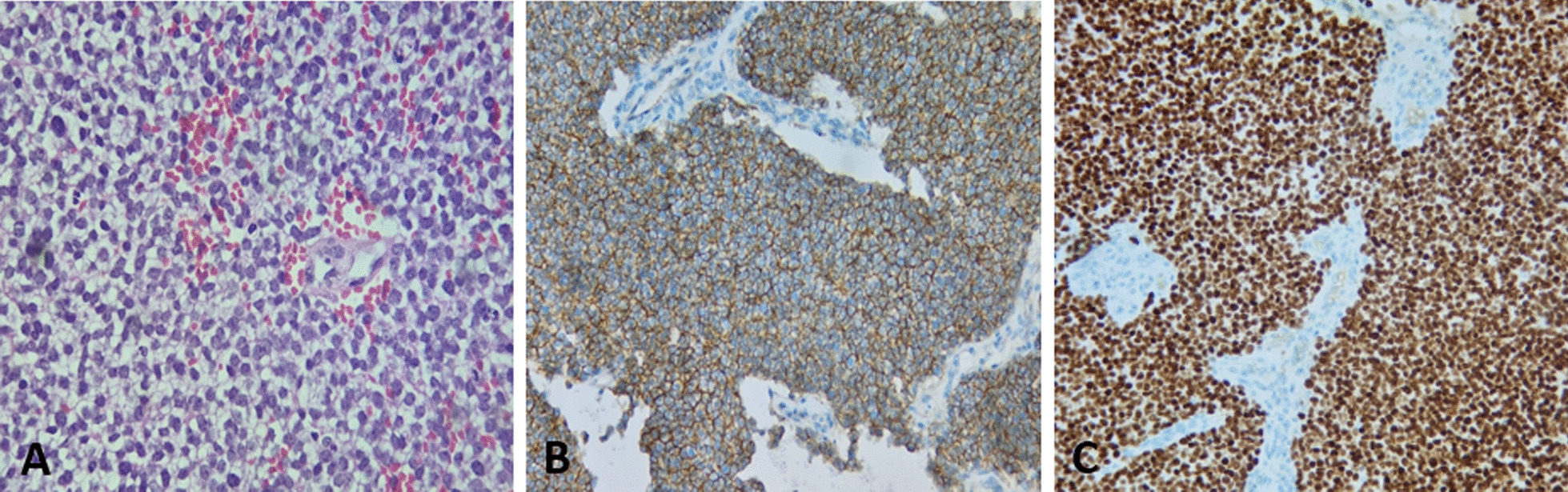
Photomicrograph of resected samples. **A** High-power image of hematoxylin and eosin stain showing uniform small round cells with fine stippled chromatin, inconspicuous nucleoli, scant clear to eosinophilic cytoplasm, indistinct cytoplasmic membranes, and focal necrosis. Immunohistochemical findings revealed positive staining for **B** CD99 and **C** NKX2.2

During hospitalization, there was a worsening of power in both feet dorsiflexion, extensor hallucis longus extension, and plantar flexion, especially on the left side with manual muscle power of 1/5. Thus, MRI of the spine and brain was done and showed heterogeneous contrast enhancement suggesting acute intratumoral hemorrhage with a subsequent increase in the cephalo-caudal dimension of the lesion, as demonstrated in Fig. 1. To prevent further neurological deficits, the patient was transferred to the neurosurgery department and underwent emergent L3–L5 laminotomy with microscopic intradural gross total resection of the tumor and laminoplasty under neuromonitoring on 4 May 2022. The amplitude of intraoperative neurological monitoring, measured using motor evoked potential test (MEPs) and somatosensory evoked potential test (SEPs), in the bilateral lower limbs’ distal muscles and sphincters improved significantly during and after tumor resection. Histopathology of the tumor showed undifferentiated small round cell sarcoma, morphologically and immunohistochemically consistent with extraskeletal Ewing sarcoma as shown in Fig. 2. Immunohistochemical studies showed that the tumor cells were positive for cluster of differentiation 99 (CD99) (diffuse, membranous) and friend leukemia integration 1 transcription factor (FLI1) (nuclear, weak), while they were negative for epithelial membrane antigen (EMA), Desmin, leukocyte common antigen (LCA), Sry-related HMg-Box gene 10 (SOX10), myogenin, and synaptophysin.

**Fig. 3:**
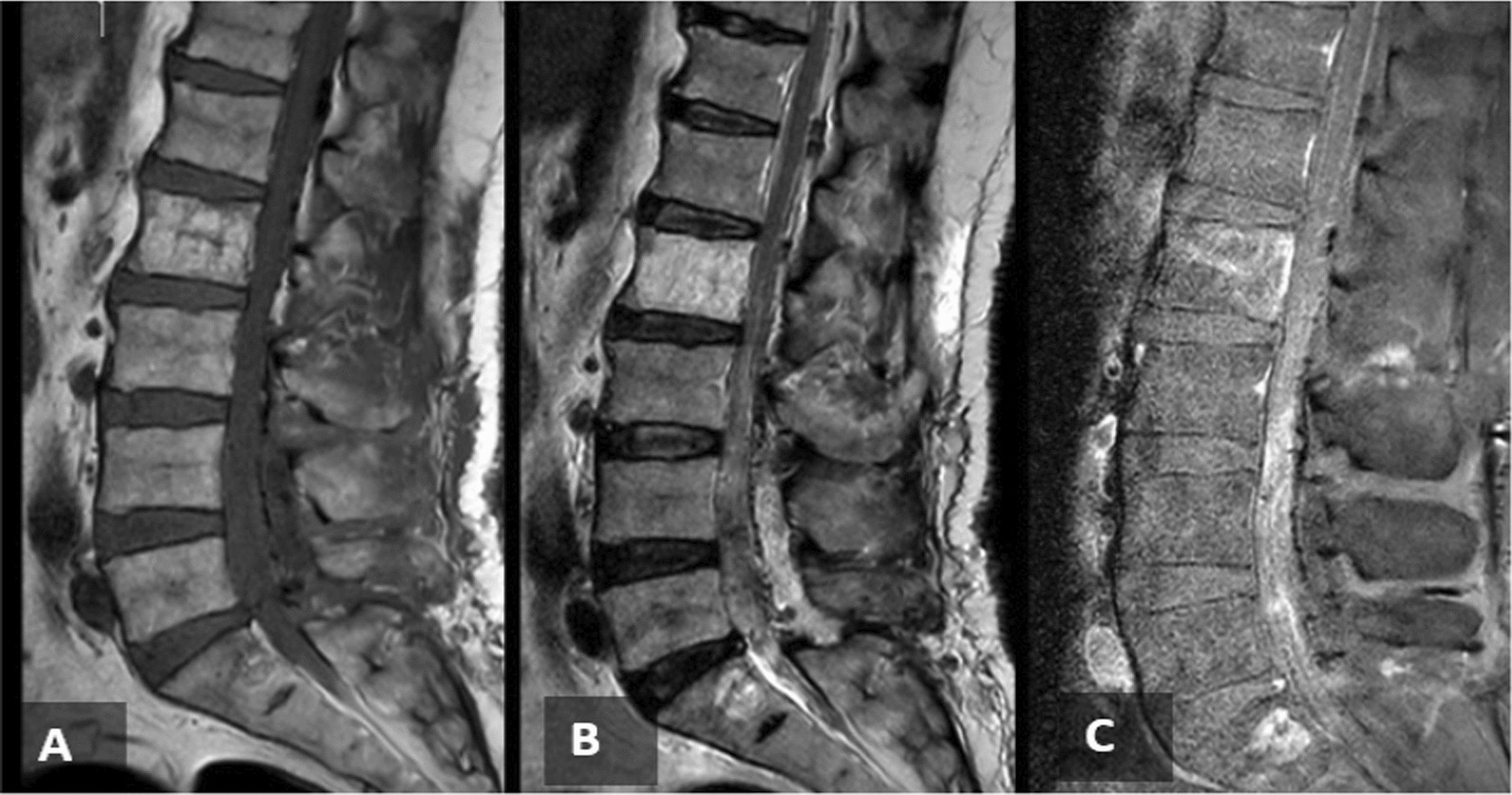
**A**, **B** and **C**, sagittal T1, T2, and T1 contrasted postoperative MRI imaging of the lumber spine respectively, showing gross total resection of the lesion with postoperative changes

Postoperative MRIs of the spine were done and revealed complete resection of the primary tumor (Fig. 3). The postoperative hospital course of the patient went smoothly with dramatic improvement of lower limb power bilaterally with dorsiflexion grade 4/5 in both limbs and intact sensations. After the surgical resection of the tumor, whole-body positron emission tomography (PET) was done and showed no evidence of local residual or distant metastasis. The patient was then supposed to start 17 cycles of a combined alternating vincristine, adriamycin, cyclophosphamide (VAC)/ifosfamide and etoposide (IE) regimen as an adjuvant treatment. However, the patient only received three cycles.

The patient had his first course of IE, followed after 21 days by VAC. Then, he was given IE again 3 weeks later. The first course of IE and the second course of VAC were administrated as full doses. However, the first one was complicated by neutropenic fever and electrolyte disturbances, while the second one, was complicated neutropenia, prolonged diarrhea, and acute kidney injury. The third course of IE was only given for 2 days instead of 5 because the patient’s status was complicated by ifosfamide-induced encephalopathy.

Thus, the oncologist decided to stop chemotherapy temporarily due to its complications and influence on the patient’s quality of life and recommended starting adjuvant radiotherapy. Accordingly, he was started on radiotherapy (total dose of 45 Gray in 25 fractions for the lumbosacral region only). Following the completion of radiotherapy on 16 November 2022, the patient’s symptoms improved, and he resumed his previous daily routine. On 12 January 2023, a comprehensive assessment using F18-fluorodeoxyglucose (FDG) PET and CT scans revealed physiologic metabolic activity in various body regions, including the brain, cervical glands, and liver. Stable mediastinal lymph nodes were observed with no significant fluorodeoxyglucose (FDG) uptake. No concerning findings were noted in the chest or abdomen/pelvis, except for mild thickening in the left adrenal gland and metabolic activity in the large bowel due to metformin consumption. Postoperative and postradiation changes were observed in the musculoskeletal system, which were related to the prior tumor resection. Overall, the assessment suggests complete metabolic remission in a known case of lumbar spine extraskeletal Ewing sarcoma, with no other concerning hypermetabolic activity detected.

During the follow-up visit on 6 June 2023, the new PET–CT scan showed a newly developed focal bone FDG lesion within the right ischium, requiring further assessment and additional follow-up to rule out serious pathology, with no evidence of other suspicious lesions in this study. On 22 August 2023, a PET–CT scan was performed to assess the progression of this patient’s tumor. The study showed resolution of the focal FDG uptake at the level of the right inferior pubic bone, along with a healing fracture seen on the corresponding CT scan. However, since 6 June 2023, there has been an increase in the FDG uptake and extent of focal spinal canal uptake at the level of L4, which is suspicious for local tumor recurrence, with no evidence of distal metastasis. The case will be discussed in the tumor board to carefully analyze the possibilities for surgery or adjuvant radio-chemotherapy.

## Discussion

This paper presents an extremely rare case of primary intradural extramedullary Ewing sarcoma (IEES). A search for cases of IEES was done from 1997 to 2022 and we found a total of 54 cases [11–53]. The present study involved a comprehensive analysis of various demographic features of the cases, including age, sex, location, clinical manifestations, adjuvant therapy, and clinical outcomes, which are concisely summarized in Table 1. Among the 54 patients whose cases were analyzed, 63% (*n* = 34) were male and 37% (*n* = 20) were female. At the time of diagnosis, the patients’ ages ranged from 3 to 70 years, with a mean age of 30.22 years and a median age of 30 years. Notably, the lumbar region was found to be the most commonly affected location (*n* = 16, 29.6%), followed by the thoracolumbar region (*n* = 15, 27.8%). The most frequently reported clinical complaint among patients was pain (*n* = 39, 72.2%). Additionally, motor impairment of either an upper or lower limb was noted in 21 patients (38.9%), while bladder and rectal disturbances were observed in eight patients (14.8%).

**Table 1:** Summary of the literature review

Characteristic		
Age (years)	Mean	30.22
	Median	30
	Range	30–70
Male	63.00%	
Primary site	Cervical	11.1%
	Thoracic	5.6%
	Lumber	29.6%
	Sacral	0
	Thoracolumber	27.8%
	Lumbosacral	16.7%
	Cervicothoracic	7.4%
	Thoracolumbosacral	1.9%
Year of diagnosis	1997–2001	9.3%
	2002–2006	9.3%
	2007–2011	25.9%
	2012–2016	33.3%
	2017–2021	20.4%
	2022	1.9%
Presentation	Pain	72.2%
	Motor deficits	38.9%
	Sensory deficits	24.1%
	Sphincter disturbances	14.8%
	NA	14.8%
Resection	GTR	44.4%
	STR	38.9%
	Biopsy	1.9%
	NA	14.8%
Adjuvant therapy	Chemotherapy alone	11.1%
	Radiotherapy alone	3.7%
	Chemoradiotherapy	63%
	No	5.6%
	NA	16.7%
CD99	Positive	83.3%
	NA	16.7%
Synaptophysin	Positive	31.5%
	Negative	14.8%
	NA	53.7%
EWSR1 gene translocations	t(11;22)	38.9%
	t(21;22)	1.9%
	Unspecified	7.4%
	Negative	1.9%
	NA	50%
Outcome	AWD	22.2%
	DOD	20.4%
	NED	35.2%
	NA	22.2%
Recurrence	Positive	38.9%
	Negative	29.6%
	NA	31.5%

*NA* not available, *GTR* gross total resection, *STR* subtotal resection, *AWD* alive with disease, *DOD* dead of disease, *NED* no evidence of disease

Our reported case of IEES was a 58-year-old Palestinian male with lumbosacral involvement. Only 16.7% of IEES cases reported in the literature presented in the lumbosacral region. The patient suffered from low back pain with radiculopathy. The history was also significant for motor and sensory deficits. Additionally, he suffered from sphincter disturbances.

The most frequent symptom related to IEES and spinal tumors, in general, is pain. They tend to progress slowly and develop symptoms over a long period of time due to neural compression. Acute presentation is very rare and has been described only in a few cases in the literature. Our patient had sudden deterioration of his motor and sensory functions due to intratumoral bleeding. This rapid onset of neurological deficit could be explained by the accelerating growth of the tumor and subsequent compression of the adjacent neural structures. The exact mechanism of intratumoral hemorrhage is still unknown. Nevertheless, many theories were stated in an attempt to explain this phenomenon. The most accepted one assumed that the rapid growth rate of the tumor and abnormal vascular proliferation might increase the fragility of the tumor’s blood vessels and increase the chance of bleeding inside the tumor [54, 55].

The diagnosis of IEES is challenging due to its rarity and the fact it presents similarly to other intradural spinal tumors such as ependymomas, astrocytomas, and meningiomas [56]. Ewing sarcoma is a poorly differentiated malignant tumor with small blue round cell under the microscope [2]. This tumor exhibits special molecular characteristics and varying neuroectodermal differentiation by immunohistochemistry. Ewing sarcoma (ES) shows immunopositivity for multiple immunohistochemical markers. Among those, CD99 is the most sensitive [57], testing positive in about 95% of EES cases [58]. All IEES cases reported in the literature with known CD99 expression showed immunopositivity for CD99 (*n* = 45). As for our case, it was also positive for CD99, which is concurrent to other cases of IEES reported in the literature. Despite its high sensitivity, CD99 is also expressed by other malignancies besides ES, such as T-lymphoblastic lymphoma and small-cell anaplastic osteosarcoma [59–62]. Thus, the use of CD99 expression by tumor cells is not sufficient to make the diagnosis of Ewing sarcoma. Many other surface markers are usually ordered to help in the diagnosis process. All these markers are less specific for ES when compared with CD99. Examples of these markers include synaptophysin, vimentin, and FLI-1. When it comes to synaptophysin, 17 cases were positive, while 8 were negative, with no available data about the rest of the cases.

In our case, immunohistochemical analysis of the tumor’s tissue showed immunopositivity for FLI1-1, while it was negative for synaptophysin. ES sarcoma has a pattern of nonrandom chromosomal translocations involving the Ewing sarcoma breakpoint region 1 (EWSR1) gene on chromosome 22 with one of the E26 transformation-specific (ETS) transcription factors. Reverse transcription polymerase chain reaction (RT–PCR) or fluorescence in situ hybridization (FISH) can be used to detect such translocations. The most frequent translocation in ES is t (11;22) (q24: q12), seen in 90% of ES cases [63] and was detected in 21 of the IEES reported cases. This results in the formation of EWS/FLI-1 fusion transcript, which plays an important role in ES pathogenesis. EWSR1 rearrangement is not necessary for the diagnosis of ES. Many reports suggest that not all ES cases have EWSR1 gene rearrangement; thus, it is not specific to Ewing sarcoma. One of the IEES cases reported by Yan *et al*. had no detected rearrangements by FISH. This may indicate that other mechanisms may be involved in the pathogenesis of ES.

ES is one of the highly aggressive bone and soft tissue tumors. It usually presents with micro or macro-metastasis at the time of diagnosis; thus, most patients die from disseminated disease without systemic multimodal treatment [64]. Despite aggressive treatment, many trials showed that this only improved the overall survival in patients with localized disease. The survival of patients with metastatic disease is still limited to 20–25% [65, 66]. Still, there is a high chance of recurrence upon the end of treatment, even in patients with primary nonmetastatic disease.

The treatment principles for EES are still similar to those of Ewing sarcoma of the bone. The surgical margin status is one of the most reliable indicators of the tumor left in the patient [67], and it affects the overall survival and the rate of recurrence [68, 69]. The margins must be wide enough to ensure optimal oncological control and narrow enough to maximize the function. Neoadjuvant treatment helps reduce the tumor size and, thus, facilitates the total resection of the tumor.

Management of Ewing sarcoma involves many cycles of systemic therapy combined with local treatment by surgery, radiation, or a combination of both in an attempt to eradicate the tumor. The use of chemotherapy has significantly improved the 5 year survival from 10% to over 70% in patients with localized disease [70]. However, it does not improve the overall survival of patients with metastatic disease. The 5 year overall survival is still limited to less than 30%. This is even worse in patients with disease relapse. Initially, chemotherapy was used in the adjuvant setting to control metastatic disease. However, it is now administered before local therapy (neoadjuvant therapy) to treat micro-metastatic disease and improve local control [71].

In the USA, standard chemotherapy for EWS includes VDC/IE, administered on an interval compressed schedule [72]. The length of adjuvant chemotherapy is determined by histopathological response to chemotherapy and the presence of metastasis at the time of diagnosis. Ewing sarcoma is considered radiosensitive [1]. Radiotherapy (RT) is used in different stages of the disease. It could be used in inoperable cases or in combination with surgery for operable ones. It also benefits patients in palliative settings [73]. Radiotherapy is usually avoided in patients with no residual disease, “resection with clean margin,” to avoid the complication of radiation. However, RT is an essential component of therapy for patients undergoing resection if the surgical margins are inadequate or in cases of metastatic disease [74, 75]. The aggressivity of tumor treatment is associated with significant psychological and physical morbidities for the survivors.

Generally, patients with spinal tumors undergo surgical resection of the tumor, either partially or globally, followed by radiotherapy and multiple cycles of chemotherapy. Although previous reports recommended that IEES should be treated with adequate cycles of intensive chemotherapy at appropriate intervals [76] and craniospinal radiotherapy to prevent recurrence or distal metastasis [40], our review of the literature revealed that only 63% of the patients received adjuvant chemoradiotherapy for the management of their disease while 11.3% received chemotherapy alone, 3.7% received radiotherapy alone, and 5.6% did not get any adjuvant therapy. Despite treatment, only 43.2% of cases with known outcomes did not get disease recurrence during their follow-up period, and 45.2% of cases with known outcomes had no evidence of disease after completing their treatment courses. These numbers indicate that IEES has a poorer prognosis when compared with osseous ES, which suggests that we need to develop a new protocol to improve the outcome and cure rates of those patients in the future.

## Conclusions

Although IEES is very rare and only a few cases have been reported, clinicians should be aware of this tumor and consider it in the differential diagnosis of spinal lesions in children and adults or those who present with back pain or radicular pain. Our case was initially assumed to be myxopapillary ependymoma tumor but was finally diagnosed as an extraskeletal Ewing sarcoma. The definitive diagnosis is made by histopathological examination and immunohistochemistry. The most effective treatment seems to be gross total resection, although adjuvant therapies such as radiation therapy and chemotherapy could improve life expectancy. Despite this, the prognosis of EES is still poorer than the osseous ES, which suggests the need for new protocols for the management of EES.

## Data Availability

The original contributions presented in the study are included in the article/Supplementary Material, further inquiries can be directed to the corresponding author/s.

## Abbreviations

ES: Ewing sarcoma
IEES: Intradural extraskeletal Ewing sarcoma
MRI: Magnetic resonance imaging
CT: Computed tomography
MEPs: Motor evoked potential test
SEPs: Somatosensory evoked potential test
CD99: Cluster of differentiation 99
FLI1: Friend leukemia integration 1 transcription factor
EMA: Epithelial membrane antigen
LCA: Leukocyte common antigen
SOX10: Sry-related HMg-Box gene 10
PET: Positron emission tomography
IE: Ifosfamide and etoposide
VAC: Vincristine, adriamycin, cyclophosphamide
EWSR1: Ewing sarcoma breakpoint region 1
ETS: E26 transformation-specific
RT–PCR: Reverse transcription polymerase chain reaction
FISH: Fluorescence in situ hybridization
NA: Not available
GTR: Gross total resection
STR: Subtotal resection
AWD: Alive with disease
DOD: Dead of disease
NED: No evidence of disease
FDG: Fluorodeoxyglucose
